# Two Cases of Sycosis

**Published:** 1895-04

**Authors:** Sarah N. Smith

**Affiliations:** New York


### TWO CASES OF SYCOSIS.

Sarah N. Smith, M. D., New York.

### Sycosis No. 1.

In May, 1893, a young lady some fifteen years old consulted me relative to a sore on her hand that was very annoying as well as very mortifying. On the back of the right hand was an ugly looking sore that discharged most of the time, but not very freely.

"How long has it troubled you?"

"For three years or more."

"What have you done for it?"

"I consulted an allopathic doctor in Albany some time since, who gave me some ointment to apply externally, saying, that if it did not help me, to return to him in two or three weeks and he would scrape it out."

"What would *you* think of scraping, as he suggests?"

"I shouldn't consider it for a moment, as I should fear that it would injure rather than benefit."

She wished me to prescribe for her, which I did reluctantly, as there were no guiding symptoms, save the appearance of the ulcer; this was nearly the size of a quarter of a dollar, with a scanty discharge; ragged, ugly looking. The only thing that I could learn was that it was very sore, smarting and burning. After careful thought, I decided that it was in the nature of a wart.

I gave her five powders of Thuja$^{200}$ (Dunham), with orders to take one every evening, and call when all were used. She came, as requested, and called out, as she entered my room, "Dr. Smith, you are curing my hand. It is ever so much better, and not sore any more."

"How long will it take to cure it?" she inquired.

"It may take a month, and perhaps longer."

"I don't care what it costs, if you will only cure it. It looks so bad that it hurts my pride."

Who could wonder! Suffice it to say, that a month did the work. Thuja$^{200m}$ was all that I gave her until the last prescription, when I gave her Silicea$^{m}$ to smooth and finish up the work.

I left town the following Tuesday. The mother sent for me the same day, as this young lady had diphtheria. When I returned on Friday she was laid away to rest, under the treatment of a genuine "regular."

### Sycosis No. 2.

Last September, after returning to New York, one of my patients came to me with a bad looking hand. She was very much alarmed about it; said that it had been there some time. It was a bunch as large as a walnut, smooth and hard, of a bright reddish purple. The only symptoms I could obtain: she said that it was very sore to the touch, and hurt and burned. I was at a loss what to christen it; but gave Lachesis$^{800}$ (Jenner), from the color, etc., rather than from symptoms. It improved the color and relieved the tenderness somewhat, but results were not satisfactory, to myself at least. I was going out-of-town and advised the patient to go to the Homœopathic Dispensary, Sixty-fourth Street and Second Avenue. I saw nothing more of her for several weeks, when she came to my office and said she had come to have her hand cured; said that it was growing worse instead of better.

In the meantime another growth had appeared close to the original one, quite similar in appearance, but not as large. The thought then came to me, that it might be a case of sycosis. Prescribed Thuja$^{200}$ for a week. When she returned in a week she said the medicine helped the sick hand at once; it was doing well now. She came two or three times after, and as the cure steadily went on, she was greatly delighted to know that she was to be relieved from such an eye-sore as she had carried all these months.

When I saw her some two months after, she said her hand was healed and her general health greatly improved.

